# Nitrogen-converting communities in aerobic granules at different hydraulic retention times (HRTs) and operational modes

**DOI:** 10.1007/s11274-014-1766-1

**Published:** 2014-11-04

**Authors:** Agnieszka Cydzik-Kwiatkowska, Irena Wojnowska-Baryła

**Affiliations:** Department of Environmental Biotechnology, University of Warmia and Mazury in Olsztyn, Słoneczna 45 G, 10-709 Olsztyn, Poland

**Keywords:** Hydraulic retention time (HRT), Bacterial activity, Anoxic/oxic mode, Aerobic granular sludge

## Abstract

This study determined how the activity and number of nitrogen-converting microorganisms varied with changes in hydraulic retention time (HRT) and the operating regime of aerobic granular sequencing batch reactors (GSBRs) treating high-nitrogen wastewater. Continuously aerated (O-mode) GSBRs were operated at HRTs of 10-, 13- and 19-h. Then the same reactors were operated at identical HRTs but the cycles started with an anoxic phase (A/O mode). To investigate the microbial communities, DNA- and RNA-based relative real-time PCR was used. In all experimental reactors ammonium was fully removed with a removal rate up to 75 mg N–NH_4_^+^/(L·h), and nitrification efficiency was above 90 %. The efficiency of the removal of oxidized nitrogen forms decreased with the lengthening of HRT. The study found that variable oxic conditions (A/O mode) in the GSBR cycle stimulated the simultaneous activity of ammonium oxidizing bacteria (AOB), N_2_O-reducers, and Anammox bacteria in aerobic granules. With both modes, the activity of nitrogen-converting bacteria was highest with a 13-h HRT. Shortening HRT, resulted in higher chemical oxygen demand and nitrogen loadings, which favored the growth of Anammox microorganisms in granules and caused a decrease in the number of AOB. With all HRTs, the number of Anammox microorganisms was about 1.5-times higher in A/O mode than in O mode.

**Electronic supplementary material:**

The online version of this article (doi:10.1007/s11274-014-1766-1) contains supplementary material, which is available to authorized users.

## Introduction

In recent years, there has been intensive development of wastewater treatment based on aerobic granular sludge cultivated in sequencing batch reactors (GSBRs). This is because aerobic granules offer several advantages when compared to activated sludge: they have greater size, more compact structure and higher resistance to organic load and toxic substances. The granules are formed by spontaneous immobilization, and are spherical with a defined, regular outer shape. Granular biomass also has a very good settling ability that helps to maintain a high concentration of biomass in the reactor (Adav et al. 2008).

For successful nitrogen removal, operational conditions of the GSBR must be carefully selected. Hydraulic retention time is one of the most important variables. If HRT is too long, energy consumption for aeration is high and the starvation phase in the GSBR cycle is long. Too short an HRT increases the risk of inefficient pollutant removal and produces a large excess of biomass as a result of a high pollutant loading.

The presence of aerobic, anoxic and anaerobic zones in the granule structure provides favorable conditions for the growth of both aerobic nitrifiers, and anaerobic and anoxic microorganisms. The presence of metabolically diverse microorganisms in the granule structure enables simultaneous removal of nitrogen and phosphorus from wastewater (Wang et al. 2009). Microbial diversity and abundance in the granules depends on operational conditions, such as the type of organic substrate, the loading rate or aeration intensity (Liu and Tay 2004). In the GSBR cycle, anoxic or anaerobic phases may increase the efficiency of nitrogen removal (Chen et al. 2011; Zhang et al. 2011). In general, full denitrification to N_2_ in aerobic granular sludge should be promoted because N_2_O induces global warming and contributes to the destruction of the stratospheric ozone layer (Kampschreur et al. 2009).

Because the biomass in wastewater treatment systems is created by multi-species microbial communities, a variety of pollutants can be removed. This complexity makes it necessary to use advanced molecular tools to study the species structure and activity of microorganisms in biofilm, activated sludge or granular sludge. Research has already shown that a few common floc-forming species of *Beta*- and *Gammaproteobacteria* and *Flavobacterium* sp. predominate in mature granules (Li et al. 2008). It has been demonstrated that the balance between nitrifying and denitrifying bacteria in granular sludge depends on the COD/N ratio in the feed (Yang et al. 2003) and that ammonium load is a major factor influencing ammonium oxidizing bacteria number and species composition in granules (Cydzik-Kwiatkowska and Wojnowska-Baryła 2011). Removal of nitrogen inside the structure of the granules can also be the result of the activity of anaerobic ammonium oxidizing bacteria (Winkler et al. 2012).

The analysis of chromosomal DNA indicates the size of the population, whereas the analysis of labile RNA, which is synthesized in response to changing environmental conditions, indicates the activity of the microorganisms in the biomass (McIlroy et al. 2009). Although DNA analysis may reveal the presence of functional genes in environmental samples, this does not mean the bacteria present in the environment will display the activities that correspond to those genes. Assays based on the 16S rRNA gene can be used to make targeted measurements of the activity of a particular group of microorganisms, whereas assays based on mRNA, which comprises up to 5 % of the total RNA pool in a cell, can be used to identify the activities of the functional genes that are associated with specific metabolic pathways.

It has been indicated by physicochemical analyses and molecular studies focused on identification of bacteria in biomass that nitrogen can be simultaneously removed with different metabolic pathways in reactors with aerobic granules. Research on the activity of groups of bacteria in aerated sequencing batch reactors with typical activated sludge has been conducted by Aoi et al. (2004) and Cydzik-Kwiatkowska et al. (2007). However, there is a lack of data on the activity of genes involved in the metabolism of nitrogen compounds in granules during the GSBR cycle and on how this activity changes with different operational conditions. Therefore, this study aimed to determine how the use of different aeration modes and HRTs affects the abundance and activity of nitrogen-converting microorganisms in aerobic granular sludge. To achieve these aims, DNA- and RNA-based relative real-time PCR was used.

## Materials and methods

### Substrate

Anaerobic digester supernatant was used as a substrate; it came from the “Łyna” municipal wastewater treatment plant in Olsztyn (Poland). The values of the pollutant concentrations were about 800 mg COD/L (ranging from 380 to 850 mg COD/L), 570 mg TKN/L (Total Kjeldahl Nitrogen/L), 470 mg N–NH_4_^+^/L, 12 mg P/L and reaction of 8.8 pH. Carbonates/carbohydrates were added to the supernatant in the concentrations theoretically required for nitrification (Villaverde et al. 1997). Since the COD in the supernatant was due to slowly biodegradable organics, sodium acetate was added to the supernatant as an external carbon source for biomass synthesis and as an electron donor for denitrification in amount of 800 mg COD/L in all experiments.

### GSBR operation

The experiment was conducted in three column-GSBRs (height 100 cm, diameter 10 cm) with a working volume of 4.5 L. The seed sludge (SS) was aerobic granular sludge cultivated on digester supernatant diluted to a 1:1 (v:v) proportion with tap water (the concentrations of TKN and COD in the mixture were about 150 mg/L). The reactors were operated at a volumetric exchange rate of 67 % per cycle, a temperature of 26 °C and a reaction pH from 7.5 to 8.5. Air was supplied with a superficial velocity of 0.8 cm/s. Initially, three constantly aerated (O mode) GSBRs were operated at cycle lengths (*t*) of 6, 8 and 12 h with corresponding HRTs of 10, 13 and 19 h. These reactors were then operated at identical HRTs but aeration was preceded by a 45-min anoxic phase at the beginning of the GSBR cycle (A/O mode). Filling, settling and decantation in the GSBR cycle lasted for 5 min. Biomass concentration in the GSBRs ranged 7–9 g TSS/L. Each experimental reactor is referred to with an abbreviation in which the first term in the subscript refers to the operational mode and the second indicates the HRT value (e.g., R_O_10h_ is a GSBR operated in O mode working at an HRT of 10 h). The operational conditions of the experimental reactors are given in Table 1. In both O and A/O mode, about 180 GSBR cycles were carried out.

**Table 1:** Operational parameters of the GSBRs

Parameter	Unit	R_O_9h_	R_O_12h_	R_O_18h_	R_A/O_9h_	R_A/O_12h_	R_A/O_18h_
Cycle length	h	6	8	12	6	8	12
HRT	h	10	13	19	10	13	19
VLR_C_	g COD/(L·d)	3.7	2.8	1.8	3.1	2.4	1.6
VLR_N_	g N/(L·d)	1.4	1.1	0.7	1.4	1.1	0.7
COD/N	–	2.6	2.6	2.6	2.2	2.2	2.2

*HRT* hydraulic retention time, *VLR*_*C*_ organics volumetric loading rate, *VLR*_*N*_ nitrogen volumetric loading rate

### Analytical methods

Wastewater and biomass in the reactors were analyzed in accordance with APHA (1992). Nitrates and nitrites were measured as mg N–NO_2_^−^ or N–NO_3_^−^/L. Measurements were carried out during the GSBR cycle to observe the changes in the nitrogen compounds’ concentrations over time. Nitrification efficiency was calculated as a percentage of oxidized nitrogen forms to TKN less the N used for biomass synthesis, while denitrification as a percentage of N reduced to all oxidized nitrogen forms in the reactor. Dissolved oxygen (DO) concentration was measured using ProODO (YSI Environmental, USA).

### Real-time PCR

For measurement of relative abundance of bacteria, granular biomass samples were taken at the end of operation in both O and A/O mode, at which time the characteristics of the effluent were stable. In addition, during the period of stable reactor operation before the end of each mode, sampling was performed during two GSBR cycles at 1–3-h intervals to investigate how the activity of the nitrogen converting bacteria changed during the cycle. Respective samples from the two cycles were mixed before isolation.

Both DNA and RNA were isolated in duplicate and then the isolates were mixed. DNA was extracted from approximately 150 mg of centrifuged sample using a FastDNA^®^ SPIN^®^Kit (Q-BIOgene, Canada). The concentration of the DNA was measured using a BioPhotometer (Eppendorf, Germany). The DNA isolated from biomass samples was of high purity and its concentrations of were in the range from 102 to 134 µg/mL. Before RNA isolation, granular sludge was fixed according to Cydzik-Kwiatkowska and Wnuk (2011). Isolation was carried out using a Total RNA kit (A&A Biotechnology, Poland). The concentration of RNA was measured using a Qubit™ fluorometer (Invitrogen, USA). The average concentrations of RNA isolated from GSBRs operated at HRTs of 10, 13 and 19 h of RNA were 606 ± 265, 1,097 ± 254, and 1,347 ± 244 µg/mL, respectively. On the basis of these concentrations, identical amounts of RNA (300 µg) were taken for reverse transcription (real-time PCR normalization). Reverse transcription was conducted with the use of a RevertAid™ H Minus First Strand cDNA Synthesis Kit (Fermentas, Canada).

Real-time PCR for a particular sample was performed in quadruplicate using the primers and thermal profiles given in Table 2. The reaction mixture for the assessment of the number of microorganisms contained: 10 µL of Power SYBR Green PCR Master Mix (Applied Biosystems), 100 nM of the particular primer, 0.25 ng DNA/µL and water for a final volume of 20 µL. The reaction mixture for the assessment of microbial activity contained: 10 µL of Power SYBR Green PCR Master Mix (Applied Biosystems), 100 nM of the particular primer, 1.5 µL of cDNA and water for a final volume of 20 µL. During *amoA* gene amplifications, 50 nM of KCl were added to increase the reaction specificity. Reactions were carried out in a 7500 Real Time PCR System (Applied Biosystems, USA). The fluorescence signal was normalized by dividing the SYBR dye emission by the reference dye (ROX) signal intensity. Data were analyzed with Sequence Detection Software, version 1.3 (Applied Biosystem). A dissociation stage was conducted after real-time amplification, to confirm the melting temperature of the PCR products. The products were also electrophoresed in the presence of a GeneRuler™ 100 bp DNA Ladder Plus (Fermentas) molecular marker to check their molecular mass.

**Table 2:** Primers and real-time PCR conditions

Primer set	Reference	Target gene	Thermal conditions
519F/907R	Stubner (2002)	16S rRNA	95 °C for 15 s, 40 cycles: 50 °C for 40 s 60 °C for 40 s
amoA1F/amoA2R	Rotthauwe et al. (1997)	*amoA*	95 °C for 15 s, 40 cycles: 52 °C for 45 s 60 °C for 45 s
amx809F/amx1066R	Tsushima et al. (2007)	16S rRNA of Anammox bacteria	95 °C for 15 s, 40 cycles: 60 °C for 1 min
nosZF/nosZR	Geets et al. (2007)	*nosZ*	95 °C for 15 s, 40 cycles: 55 °C for 45 s, 60 °C for 45 s

The level of investigated DNA in seeding sludge had been assigned a value of 1 and was used as a reference during the investigations of the relative number of microorganisms in the granular sludge. The expression of the investigated RNA in granular biomass sampled from R_O_9h_ in 0.5 h of the GSBR cycle had been assigned a value of 1and was taken as a reference for the activity investigations. For the calculations of relative DNA and RNA levels, the 2^−ΔΔCt^ method was used (Livak and Schmittgen 2001).

### Statistics

The molecular results for a sample are an average of four repetitions. The relationships between individual results were determined using Pearson’s correlation coefficient. The data analysis was performed using STATISTICA 9.0 (StatSoft, USA). A value of *p* ≤ 0.05 was defined as significant.

## Results

### Technological results

**Fig. 1:**
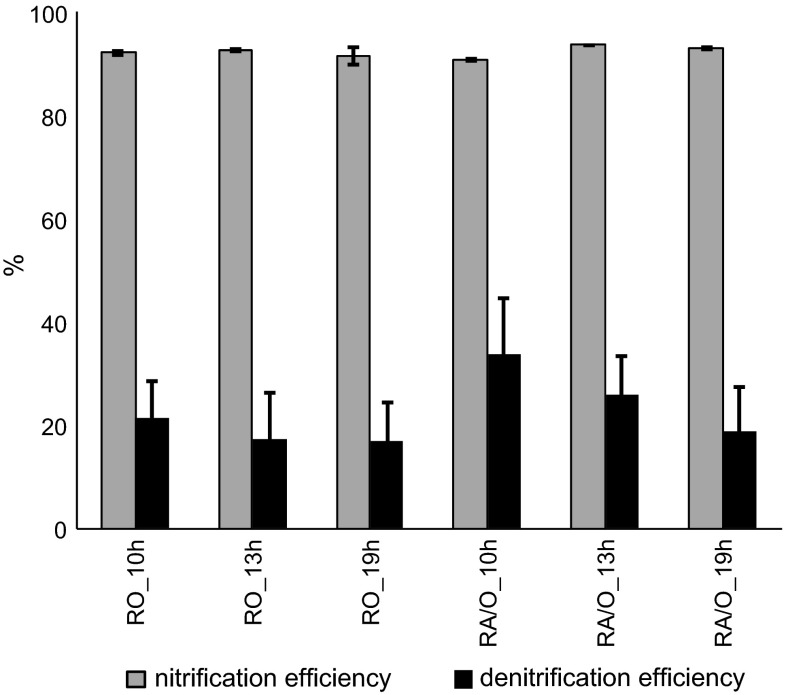
Efficiency of nitrification and denitrification in the experimental reactors

The efficiency of nitrification in all experimental reactors was higher than 90 % (Fig. 1). Denitrification efficiency was, however, influenced by both HRT and operational mode. With increasing HRTs, denitrification efficiency decreased; it was about 12, 8 and 2 % higher in the A/O-mode reactors than in the O-mode reactors. In all experimental reactors, ammonium was fully removed by the granules within the first 5 or 4 h of the cycle in O or A/O mode, respectively, which corresponded to average ammonium removal rates of 65 and 75 mg N–NH_4_^+^/(L·h). Full nitrification proceeds with nitrates as final products, however, under particular conditions e.g., high nitrogen loading or high temperature partial nitrification to nitrites can be observed. In our study, in constantly aerated reactors, the main nitrification product was nitrite; its concentration was about 400 mg/L in R_O_10h_ and R_O_13_, with a nitrates concentration below 10 mg/L. In R_O_19h_, nitrites concentration in the effluent was 350 mg/L, and nitrates concentration increased to about 30 mg/L. In R_A/O_10h_, mostly nitrites were found in the effluent (about 300 mg/L); in R_A/O_13h_, nitrite and nitrate concentrations at the end of the cycle were 220 and 100 mg/L, respectively; whereas in R_A/O_19h_, both remained at 150 mg/L.

**Fig. 2:**
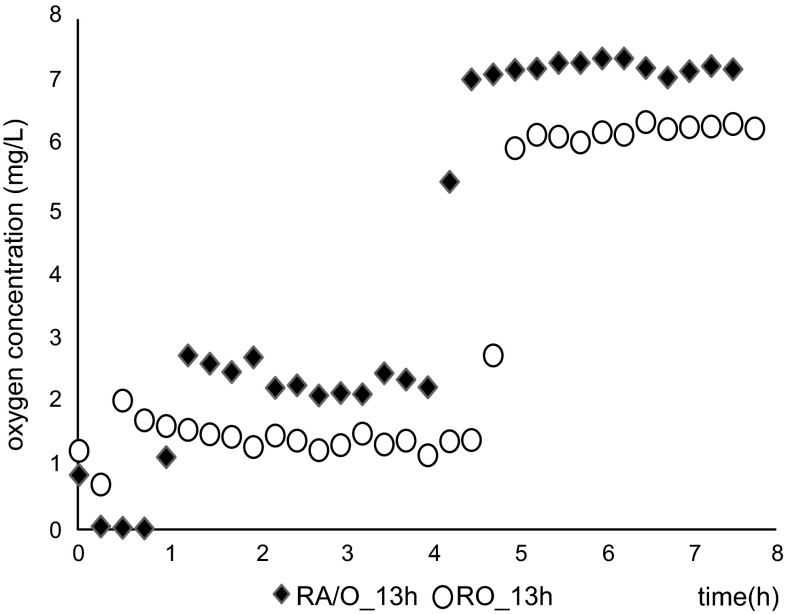
Changes in dissolved oxygen concentration during the cycle of the GSBRs operated at an HRT of 13 h

With each aeration mode, the dissolved oxygen (DO) concentrations in the cycles were similar at all HRTs, but the DO profiles of O and A/O mode GSBRs differed (Fig. 1 SM). The results for the GSBRs operated at an HRT of 13 h (cycle length 8 h) can be seen in Fig. 2. There are two differences between the modes that should be noted. Firstly, from shortly after the end of the anoxic phase in A/O mode, the DO concentration was about 1 mg/L higher than in O mode. The second difference is that the sharp increase in DO concentration, which indicates complete ammonium oxidation, occurred sooner with A/O mode.

### Abundance of microorganisms in aerobic granules

**Fig. 3:**
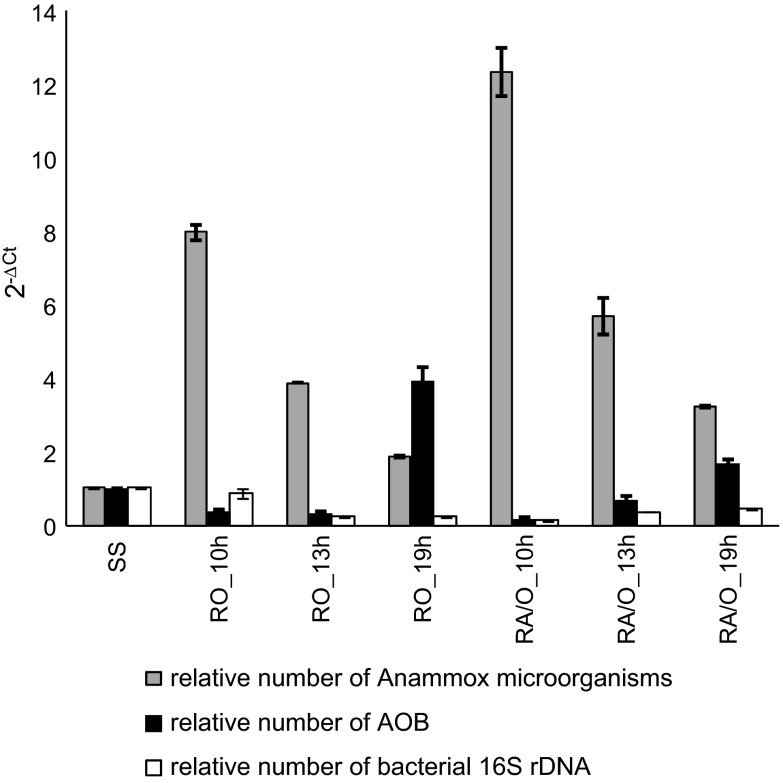
Relative number of different bacterial groups in granular sludge; SS is the reference

The shorter HRTs, which resulted in higher volumetric nitrogen and organics loading, increased the number of Anammox bacteria (R = −0.83, *p* < 0.05). When supernatant was introduced to the GSBRs operated in O mode, the number of Anammox microorganisms in the biomass increased relative to the seed sludge (Fig. 3). This increase ranged from twofold at the longest HRT, up to eightfold at an HRT of 10 h. For A/O mode GSBRs, increases in relative Anammox bacteria number were even more notable. At an HRT of 19 h, the abundance of investigated bacteria was 3-times higher, and at an HRT of 10 h,13-times higher than in the seed sludge.

In contrast, the number of AOB increased with the lengthening of HRT (R = 0.83, *p* < 0.05). At HRTs of 10 and 13, AOB number in granules was lower than in reference biomass; only at the longest HRT did it increase 4-times (O mode) and 2-times (A/O mode) in comparison to the seed sludge. At the tested HRTs, changing the mode from O to A/O increased the number of Anammox bacteria in granules about 1.5-times. Feeding the GSBRs with supernatant reduced the number of total bacteria when compared to the reference (Fig. 3). The lowest number of 16S rRNA gene was noted in the granules taken from the reactor operated in A/O mode at an HRT of 10 h.

### Activity of bacteria in the GSBR cycle

**Fig. 4:**
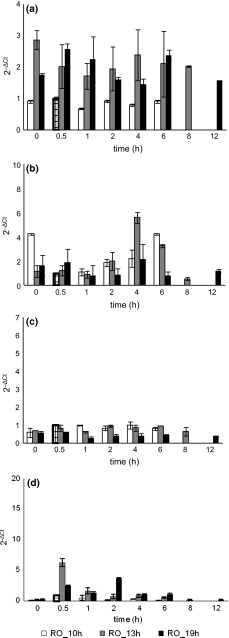
Relative level in granular sludge during the cycle of GSBRs operated in O mode of **a** 16S rRNA, **b** *amoA* mRNA, **c** 16S rRNA of Anammox bacteria, **d** *nosZ* mRNA; reference samples are *squared*

**Fig. 5:**
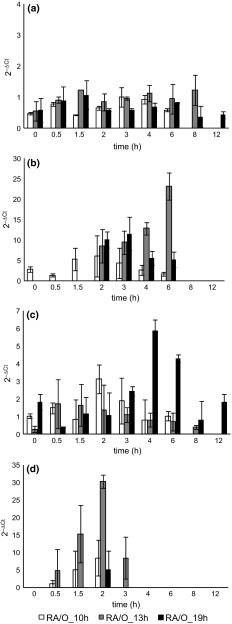
Relative level in granular sludge during the cycle of GSBRs operated in A/O mode of **a** 16S rRNA, **b** *amoA* mRNA, **c** 16S rRNA of Anammox bacteria, **d** *nosZ* mRNA

In the reactor operated in O mode at the shortest HRT, the level of bacterial 16S rRNA was relatively stable during the cycle and did not pass the value of the reference. In the biomass from the two reactors operated in the same mode but with longer HRTs, the 16S rRNA level was about 2-times higher (Fig. 4a). After the introduction of an anoxic phase, bacterial activity in R_A/O_10h_ decreased slightly, while in the two other reactors it decreased 2- to 3-fold in comparison with O mode (Fig. 5a). In all reactors, the levels of investigated RNA in the biomass peaked in the first hours of the cycle. In all reactors except R_O_10h_, 16S rRNA levels peaked again later in the cycle: in R_O_13h_ at 4 h, in R_O_19h_ at 6 h, in R_A/O_10h_ at 3 h, in R_A/O_13h_ at 8 h, and in R_A/O_19h_ at 6 h.

In constantly aerated GSBRs, *amoA* gene mRNA was present in the biomass throughout the whole cycle (Fig. 4b). The greatest fluctuations of *amoA* mRNA were in the GSBR operated at an HRT of 13 h—after 4 h of aeration, the mRNA level was 6-times higher than in the reference. In the 5th hour of the cycle, ammonium nitrogen in wastewater was depleted, which caused gradual decrease in *amoA* mRNA level in biomass. In the A/O mode reactor operated at an HRT of 10 h, *amoA* mRNA was present in biomass throughout the cycle and its transcription peaked 2 h after the cycle began. In the other A/O mode reactors, the presence of *amoA* transcripts was observed for the first time in the 2nd hour of the cycle (Fig. 5b); their number peaked in the 6th hour at an HRT of 13 h, and in the 3rd hour at an HRT of 19 h. After these points in the cycle, *amoA* mRNA was no longer detected.

In O mode reactors, the level of expression of Anammox bacteria 16S rRNA remained relatively stable during the cycle. Expression of this gene was highest in the reactor operated at the shortest HRT (Fig. 4c). The use of A/O mode led to notable increases in the expression of Anammox bacteria 16S rRNA during the cycles. At the two shorter HRTs, the highest levels of investigated RNA were noted in the first hours of the cycle (Fig. 5c). In the GSBRs operated at an HRT of 19 h, the highest activity of Anammox bacteria was observed in biomass despite their low relative abundance compared to other A/O reactors. In the 4th hour of the cycle it was more than 10-times higher than the values noted at this HRT in the constantly aerated reactor.

Despite the constant aeration of GSBRs in O mode, N_2_O-reducing bacteria in the granules were active through most of the cycle with the highest activities noted just after feeding. The highest activity was observed in the reactor operated at an HRT of 13 h; 0.5 h after the beginning of aeration the number of *nosZ* gene mRNA increased 6-times in comparison with the reference sample (Fig. 4d). The expression profiles of *nosZ* gene in biomass in the reactors operated in A/O mode are shown in Fig. 5d. At 10 and 13 h HRTs, the activity of N_2_O reducers was initially noted 0.5 h after the beginning of the cycle, peaking in the 2nd hour. In both reactors the amounts of *nosZ* mRNA were 5–8 times higher than in those reactors operated at the same HRT but in O mode. At the longest HRT, the activity of N_2_O denitrifiers was observed only in the 2nd hour of the cycle.

## Discussion

There are reports in the literature on full scale aerobic granular sludge wastewater treatment plants treating municipal wastewater or wastewater from food industry (Li et al. 2014; Giesen et al. 2013). There is, however, no information on a full scale application of aerobic granules for the treatment of high-nitrogen wastewater, therefore it is important to further optimize the process in laboratory scale. This study analyzed the relations between different HRTs and operational modes (oxic mode, anoxic/oxic mode) and the activity of nitrogen-converting bacteria, which influences the effectiveness of nitrogen conversions in aerobic granular sludge. The study found that nitrifiers, denitrifiers and Anammox bacteria were simultaneously active in aerobic granules. Their activity, including that of nitrifiers, was increased by the introduction of an anoxic phase at the beginning of the GSBR cycle while shortening HRT-induced nitrogen removal in the reactors.

In the present study, the final products of nitrification depended on the operational conditions of the GSBR and were in agreement with the observations of Belmonte et al. (2009), in that a longer hydraulic retention time stimulates full nitrification. Our research also showed that full nitrification was induced by incorporation of an anoxic phase in the GSBR cycle. According to Dytczak et al. (2008), alternating oxic conditions may promote the proliferation of fast-growing, nitrifying r-strategist bacteria such as *Nitrobacter* sp. *Nitrobacter* sp. is known to be resistant to high concentrations of nitrite, free ammonia and free nitrous acid, and to compete well with other nitrite-oxidizing bacteria at elevated oxygen and nitrite concentrations (Vadivelu et al. 2007). This may explain the improvement of nitrite oxidation in GSBRs in A/O mode. The higher nitrate production could also have resulted from higher Anammox activity in the GSBRs with an anoxic phase in the cycle.

Use of A/O mode enabled not only energy savings due to the lack of aeration during the anoxic phase, but also resulted in increased oxygen concentrations during the reactor cycle. From shortly after the end of the anoxic phase in A/O mode, the DO concentration was about 1 mg/L higher than in O mode. The higher oxygen concentration during aeration in A/O mode reactors was probably due to the fact that organics are intensively used for denitrification in the anoxic phase. The lower organic carbon content of the wastewater during the aeration phase reduced the metabolic activity of heterotrophic microorganisms in the granules, which resulted in a higher DO concentration in the bulk liquid. Also the sharp increase in DO concentration, which indicates complete ammonium oxidation, occurred sooner with A/O mode. Ammonium uptake for biomass synthesis in anoxic conditions is lower than in oxic conditions. The approximately 15 % higher ammonium removal rates with A/O mode have been therefore due to higher simultaneous activity of the AOB and Anammox bacteria that co-existed in granules.

The A/O mode and the shorter HRT promoted the predominance of Anammox bacteria in granular biomass. At each tested HRT, changing the mode from O to A/O increased the number of Anammox bacteria in granules about 1.5-times. Anammox bacteria are not able to compete for ammonium as effectively in O mode as in A/O mode reactors because the constant aeration favors nitrification. With an anoxic phase in the cycle, Anammox bacteria are more active as shown by both molecular activity measurements and higher concentrations of nitrates in the treated wastewater. Though shorter HRT increased the number of Anammox bacteria it must be remembered that HRTs cannot be shortened too much; Tang et al. (2011) reported that operating an Anammox upflow anaerobic sludge bed reactor with a short HRT lowered treatment efficiency. The adverse tendency of increasing the number with the lengthening of HRT (R = 0.83, *p* < 0.05) was observed for of AOB. *Nitrosomonas* sp. and *Nitrosospira* sp. were identified in activated sludge treating anaerobic digester supernatant (Cydzik-Kwiatkowska et al. 2012) and species of *Nitrosospira* sp. turned out to be susceptible to various HRTs. Bacteria belonging to *Nitrosospira* sp. prefer lower ammonium concentrations so a decrease in ammonia load with increasing HRT have probably favored growth *Nitrosospira*-like species that increased the abundance of AOB in the biomass.

Most bacteria possess at least a few copies of 16S rRNA (Klappenbach et al. 2001) and changes in the levels of 16S rRNA can be used to infer changes in bacterial metabolic activity in response to changes in the environment. The lowest level of 16S rRNA genes in the biomass from R_A/O_10h_ can be explained by the highest abundance of Anammox bacteria in granules under the operational parameters applied. At least some species of Anammox bacteria, as shown by the sequenced genome of “*Candidatus* Kuenenia stuttgartiensis” (Strous et al. 2006), have only one copy of 16S rRNA gene. Analysis showed that in all reactors except from R_O_10h_, the levels of investigated RNA in the biomass peaked at the beginning and in the final hours of the cycle. In these reactors, the first maxima was connected with changes in bacterial activity related to high substrate availability; the second, to activity related to the “famine” phase in the GSBR cycle and increased activity of autotrophic microorganisms.

Activity of AOB in granules was many times higher with A/O mode than with O mode, though the relative number of AOB in biomass was similar or lower than in biomass from the GSBRs operated in O mode. The applied operational parameters also shaped the activity patterns of AOB in granular sludge in the GSBR cycle. In constantly aerated GSBRs, AOB activity was recorded throughout the whole cycle. This result is similar to the results for activated sludge in a constantly aerated SBR (Cydzik-Kwiatkowska et al. 2007). Introduction of anoxic phase at HRTs of 13 and 19 h, changed the activity patterns of ammonia oxidizing bacteria—they started to be active in the 2nd hour of the cycle, and shortly after their activity decreased. In these two reactors, the timing of the appearance of *amoA* mRNA is similar to what was observed in activated sludge transferred from anaerobic to aerobic conditions (Aoi et al. 2004).

In O mode reactors, the activity of Anammox bacteria remained relatively stable during the cycle while in A/O mode the activity of these microorganisms increased and also dynamically changed in the GSBR cycle. Shortening HRT, stimulated the activity of Anammox bacteria, especially in the first hours of the cycle, which can be explained by both a lack of DO in the reactor, which promotes anaerobic ammonium oxidation, and a high concentration of substrates, including acetate. Strous et al. (2006) and Kartal et al. (2007) have shown that Anammox bacteria are facultative chemoorganotrophs and can co-metabolize organic compounds with ammonium nitrogen as a preferred electron donor.

The production of denitrification enzymes, including N_2_O reductase, can be inhibited by the presence of oxygen in the environment (Korner and Zumft 1989). It has also been observed that nitrous oxide reductase is inhibited by high nitrite concentration (Zhou et al. 2008). In our study, the observed activity of N_2_O-reducers even in O mode reactors shows that the structure of granules (in both O mode and A/O mode reactors granule diameters were similar and around 1 mm) enables complete denitrification in the deeper layers of granules despite high DO and nitrite concentrations of 350–400 mg/L. Constant aeration may also promote the appearance of denitrifiers that are capable of aerobic denitrification because they possess denitrification enzymes that are active even in oxic conditions (Beller et al. 2006; Chen et al. 2008). These bacteria could have also contributed to reduction of oxidized nitrogen forms throughout the cycle in O mode GSBRs.

Even a short anoxic phase in the GSBR cycle significantly increased the transcription of *nosZ* mRNA; however, the transcripts were present in biomass only in the initial hours of the cycle and for a much shorter time than in constantly aerated reactors. Lee et al. (2010) have shown that the use of alternating aerobic and anaerobic conditions causes occurrence of diauxic phase in denitrifying bacteria, which is required for re-synthesis of nitrate reductase enzymes and other denitrification enzymes. Denitrification depends on organic carbon presence as donors for the reduction of oxidized nitrogen forms. At HRT of 19 h the organics loading was lowest in comparison with other HRTs that may explain the shortest activity of N_2_O-reducers in the biomass in A/O reactors. From the 4th hour of the cycle no investigated mRNA was observed in the biomass from the reactors operated in A/O mode. Changes in expression patterns of the *nosZ* gene between O mode and A/O mode reactors can be explained by the species composition of biomass. In A/O mode reactors, intensive growth of anoxic denitrifiers was promoted by the anoxic conditions. Since denitrification activity is inhibited by the presence of oxygen, these bacteria were active only at the beginning of the cycle when the organics were present and the DO was low. Increase in DO up to 6–7 mg/L in the later hours of the cycle supported the diffusion of oxygen into granule structure and inhibited denitrifiers activity.

## Conclusions

This study demonstrated that:

- AOB, Anammox bacteria and N_2_O-reducing bacteria were simultaneously active in aerobic granules with diameters of about 1 mm even under constant aeration,
- incorporating even a short anoxic period in the GSBR cycle increased the activity of nitrogen-converting microorganisms, especially N_2_O reducers; their activity was many times higher than in constantly aerated reactors but lasted for shorter periods of time,
- as HRT increased, the number of Anammox microorganisms in granules sharply decreased and the number of AOB simultaneously increased;
- with A/O mode, the number of Anammox microorganisms in the biomass was about 1.5-times greater than with O mode, favoring nitrogen removal as N_2_.

## Electronic supplementary material

Below is the link to the electronic supplementary material.

Supplementary material 1 (DOCX 48 kb)
