# Supplementary material for: Nitrogen-converting communities in aerobic granules at different hydraulic retention times (HRTs) and operational modes
Source: World J Microbiol Biotechnol. 2014 Nov 4;31(1):75–83. doi: 10.1007/s11274-014-1766-1 (PMC4282690; doi:10.1007/s11274-014-1766-1)
Supplement: Supplementary file 1 — Supplementary material 1 (DOCX 48 kb) [file 11274_2014_1766_MOESM1_ESM.docx]

**Nitrogen-converting communities in aerobic granules at different hydraulic retention times (HRTs) and operational modes**

Agnieszka Cydzik-Kwiatkowska*, Irena Wojnowska-Baryła

Department of Environmental Biotechnology, University of Warmia and Mazury in Olsztyn, Słoneczna 45 G, 10-709 Olsztyn, Poland

*Correspondence: Agnieszka Cydzik-Kwiatkowska, Department of Environmental Biotechnology, University of Warmia and Mazury in Olsztyn, Słoneczna 45 G, 10-709 Olsztyn, Poland. Phone: +48 89 5234194; fax: +48 89 5234131; e-mail: [agnieszka.cydzik@uwm.edu.pl](mailto:agnieszka.cydzik@uwm.edu.pl)

**Supplementary material**

Fig. 1 SM Changes in dissolved oxygen concentration during the cycle of the GSBRs operated in O mode.
